# In vitro assessment of thyroid peroxidase inhibition by chemical exposure: comparison of cell models and detection methods

**DOI:** 10.1007/s00204-024-03766-7

**Published:** 2024-05-25

**Authors:** Runze Liu, Jiří Novák, Klára Hilscherová

**Affiliations:** RECETOX, Faculty of Science, Masaryk University, Kamenice 753/5, Pavilion A29, 625 00 Brno, Czech Republic

**Keywords:** Thyroid, Peroxidation, In vitro, Amplex UltraRed, Luminol, Cross-species

## Abstract

Disruption of the thyroid hormone (TH) system is connected with diverse adverse health outcomes in wildlife and humans. It is crucial to develop and validate suitable in vitro assays capable of measuring the disruption of the thyroid hormone (TH) system. These assays are also essential to comply with the 3R principles, aiming to replace the ex vivo tests often utilised in the chemical assessment. We compared the two commonly used assays applicable for high throughput screening [Luminol and Amplex UltraRed (AUR)] for the assessment of inhibition of thyroid peroxidase (TPO, a crucial enzyme in TH synthesis) using several cell lines and 21 compounds from different use categories. As the investigated cell lines derived from human and rat thyroid showed low or undetectable TPO expression, we developed a series of novel cell lines overexpressing human TPO protein. The HEK-TPOA7 model was prioritised for further research based on the high and stable TPO gene and protein expression. Notably, the Luminol assay detected significant peroxidase activity and signal inhibition even in Nthy-ori 3-1 and HEK293T cell lines without TPO expression, revealing its lack of specificity. Conversely, the AUR assay was specific to TPO activity. Nevertheless, despite the different specificity, both assays identified similar peroxidation inhibitors. Over half of the tested chemicals with diverse structures and from different use groups caused TPO inhibition, including some widespread environmental contaminants suggesting a potential impact of environmental chemicals on TH synthesis. Furthermore, in silico SeqAPASS analysis confirmed the high similarity of human TPO across mammals and other vertebrate classes, suggesting the applicability of HEK-TPOA7 model findings to other vertebrates.

**Supplementary Information:**

The online version contains supplementary material available at 10.1007/s00204-024-03766-7.

## Introduction

Humans and wildlife are exposed to a wide spectrum of natural and synthetic chemicals, often present in complex mixtures, that can disrupt biological processes and have adverse health effects even at low concentrations. Endocrine disruptors (EDs) are a significant category of these chemicals, capable of interfering with the synthesis, secretion, transport, binding, or regulation of hormones required for maintaining homeostasis, reproduction, development, or well-functioning metabolism (Keane et al. 2015). Within the broad spectrum of EDs, thyroid hormone disruptors (TDs) are a particularly influential subgroup that interfere with the thyroid hormone (TH) regulatory system. The TH system is regulated by the hypothalamic–pituitary–thyroid axis and plays a crucial role in various vital physiological processes, including early development, brain development, basal metabolic rate, and growth in various species (Street et al. 2018).

Disruption of the TH system can adversely affect both wildlife and humans. In children, exposure to TDs is often associated with neurodevelopmental problems, leading to cognitive and behavioural defects, lower IQ, and other challenges (Murthy and Murthy 2012). In addition, TD exposure has been linked to metabolic and cardiovascular dysfunctions, such as hypothyroidism and increased cardiovascular risk (Murthy and Murthy 2012). TDs have been also associated with obesity and diabetes (Biondi et al. 2019; Legler et al. 2015) and in both rats and humans, hypothyroidism has been connected to neurological disorders (Kakked et al. 2013; Stoica et al. 2007). Human exposure to TDs can occur through various everyday products, including personal care items, consumer products, plastics, and toys (Giulivo et al. 2016). Furthermore, environmental stressors disrupting TH regulation in aquatic vertebrates can result in adverse effects, such as increased larval mortality, suppressed sperm production, diminished egg production, impaired gonad development, hindered ovarian growth, altered swimming activity, compromised fertilisation, swim-bladder inflation in fish, and metamorphosis in amphibians (Noyes et al. 2019; Nugegoda and Kibria 2017). Thus, it is crucial to understand and minimise exposure to TDs to protect health and ecosystems.

However, despite the critical need to evaluate the potential of exposure chemicals to disrupt TH, validated high throughput methods for TDs identification are lacking. Currently, the OECD framework heavily relies on time-consuming and ethically concerning in vivo animal tests to identify adverse effects induced by TDs (Bernasconi et al. 2023; Holbech et al. 2020; Knapen et al. 2020). Thus, it is essential to develop in vitro high throughput bioassays targeting relevant mechanisms of TH system disruption, reducing reliance on animal testing. The European Commission’s European Union Reference Laboratory for Alternatives to Animal Testing (EURL ECVAM) launched a validation study to validate thyroid-relevant mechanistic methods for potential integration into the OECD test guideline framework, following OECD’s 2014 scoping document on in vitro methods for investigating TH signalling modulators (Bernasconi et al. 2023; OECD 2014). Despite these recent advances, new technologies struggle to identify thyroid-affecting chemicals due to the gap between molecular-level effects and traditional endpoints like serum TH levels and developmental neurotoxicity. The thyroid Adverse Outcome Pathway (AOP) network outlined by Noyes et al. (2019) holds promise in addressing this gap by correlating chemical targets with downstream adverse effects, thereby facilitating the identification of substances impacting thyroid activity and supporting more robust regulatory decision-making processes. One of the identified key cross-species-relevant molecular-initiating events with known adverse outcome is the inhibition of thyroid peroxidase (TPO), a critical enzyme in TH synthesis located at the apical membrane of follicular thyroid cells. Inside the follicle lumen, TPO catalyses the oxidative coupling of iodide to tyrosyl residues in thyroglobulin (TG), forming mono- and diiodinated tyrosyl residues that combine to create TH, including thyroxine (T4) and triiodothyronine (T3). TPO activity is essential in this process, and the hydrogen peroxide, necessary for the enzymatic reaction is generated through a flavoprotein-containing system (Gillam and Kopp 2001; Rokita et al. 2010).

The US Environmental Protection Agency (EPA) ToxCast program evaluated over 1000 chemicals for TPO inhibition through Amplex UltraRed (AUR) TPO assay. However, the TPO source for the study were ex vivo rat thyroids (Friedman et al. 2016) that do not comply with 3Rs principles (Replacement, Reduction, and Refinement). Moreover, TPO inhibition was elicited by the environmental pollutant mixtures in the surface water samples using the same method (Leusch et al. 2018). To minimise the reliance on animal and ex vivo material testing and adhere to the 3Rs principles, the development of sensitive in vitro based HTS methods is crucial. These methods would enable the efficient screening of chemicals without the need for extensive animal experimentation or the use of animal tissues, promoting more ethical and humane approaches in scientific research. A few previous studies have reported on in vitro high throughput amendable assays for the assessment of TPO inhibition, but they are not yet included in official test guidelines. Several studies assessed TPO inhibition caused by chemicals using luminescence emitted by the reaction of Luminol and hydrogen peroxide catalysed by TPO (Dong et al. 2020; Godlewska et al. 2018; Jomaa et al. 2015). Besides this method, several other studies utilised the detection of fluorescence emitted by Amplex UltroxRed oxidised from AUR by hydrogen peroxide in TPO-catalysed reaction, or colorimetric guaiacol (GUA) oxidation assay for measuring TPO activity inhibition (Jomaa et al. 2015; Schmutzler et al. 2007). However, there is no systematic study comparing the differences of methods and models used to evaluate TPO specificity so far.

Our research is a part of EU H2020 project ERGO focused on establishing a network of adverse outcome pathways for TH disruption using cross-vertebrate data, refining biomarkers and testing methods, for better predicting human health impacts (Holbech et al. 2020). The project namely aims to advance testing techniques and provide important information and data towards minimization of animal testing and expanding the chemical screening capabilities. In support of the undergoing international activities towards the development and validation of in vitro methods focusing on different TH system disruption modes of action we have characterized and compared the suitability of different cell lines for TPO inhibition assessment and the specificity of the TPO inhibition detection methods. The primary goal of this study is to evaluate various approaches and in vitro models for assessing the TPO activity and its inhibition by compounds or mixtures. The initial step involves a comprehensive characterization of selected cell lines and two detection methods applicable for high throughput screening (HTS), namely the Luminol (Lumi) assay and AUR assay and their specificity for TPO assessment. The GUA method was not chosen due to previous evidence of its lower sensitivity and higher TPO protein requirements compared to the AUR method (Friedman et al. 2016; Paul et al. 2014). Furthermore, our objective is to develop a highly sensitive cell line for human TPO inhibition assessment and optimise the assay conditions, as recommended by the OECD guidance on good in vitro method practices (OECD 2018), to enhance the specificity and throughput of TPO inhibition assessment. In addition, we describe the cross-species relevance potential of this endpoint by utilizing an in silico tool to compare human TPO with corresponding enzymes in other species.

## Material and methods

### Chemicals

The set of 21 test compounds in this study was chosen to address different modes of action of disruption of TH system for the use within the ERGO H2020 project (Holbech et al. 2020). Next to the two thyroid hormones, the model compounds represent a diverse set of human exposure-relevant chemicals, including different types of pharmaceuticals, pesticides, industrial chemicals, environmental contaminants, personal care and consumer products and disinfectants and antiseptics, which were obtained at high purity (≥ 98%) from Sigma-Aldrich. For further details, including CAS number and structure, see Tables 1 and S1 in Supplementary Materials. The criteria for prioritizing these chemicals for in vitro assays included their known in vivo effects, utilization in in vivo testing within the project, alignment with the EURL ECVAM validation study (Bernasconi et al. 2023), and their relevance to human-exposure scenarios.

**Table 1:** Characterization of peroxidation inhibition effects expressed as IC_50_ or IC_20_ values (*n* ≥ 3) of tested chemicals detected by AUR or Lumi assays in the current study in comparison with available previously reported data

Abbrev	Chemicals	Chemical type category	Lumi assay IC_50_ (µM)						AUR assay IC_50_ (µM)			
			Current study					Jomaa et al. (2015)	Current study	Jomaa et al. (2015)	Dong et al. (2020)	Dong et al. (2020)
			Nthy-ori 3-1	FRTL-5	HEK-TPOA7	HEK293T	ML-1^c^	Nthy-ori 3-1	HEK-TPOA7	Nthy-ori 3-1	CHO-TPO	LentiX-TPO
BP2	2,2′-4,4′-tetrahydroxy benzophenone	Personal Care Products	38.5 (± 0.79)	29.6 (± 13)	20.2 (± 5.09)	–	–		0.56 (± 0.06)		0.2	0.24
BPA	Bisphenol A	Industrial Chemical	120 (± 6.2)	143 (± 17)	130 (± 17)	122 (± 32)	142		10.2 (± 4.4)			
MMI	Methimazole	Pharmaceuticals	3.91 (± 0.67)	3.69 (± 0.70)	3.24 (± 0.63)	–	3.23	4	1.50 (± 0.39)	2.7	0.49	0.23
PFOA	Perfluorooctanoic acid	Perfluorinated Chemicals	NA	NA	110 (± 19)^a^	–	–		5.15 (± 1.5)^a^			
PFOS	Perfluorooctane sulfonate	Perfluorinated Chemicals	NA	NA	NA	–	–		232 (± 23)^a^			
PTU	6-propylthiouracil	Pharmaceuticals	11.7 (± 1.3)	13.2 (± 1.0)	8.53 (± 1.1)	18.9 (± 4.6)^b^	17.7	16.4	5.36 (± 0.87)	35.2	4.9	10.4
RSC	Resorcinol	Personal Care Products	13.8 (± 1.9)	15.7 (± 2.7)	17.9 (± 1.0)	–	17.4		0.358 (± 0.16)		0.05	0.06
SMX	Sulfamethoxazol	Antibiotics and Antimicrobials	NA	NA	NA	–	–		101 (± 14)^a^			
T3	3,3′,5-Triiodo-L-thyronine	Thyroid Hormone	NA	NA	NA	–	–		10.8 (± 1.0)^a^			
TBBPA	Tetrabromobisphenol A	Industrial Chemicals	77.2 (± 1.4)	130 (± 6.9)	90.9 (± 20)	64.4 (± 6.7)	–		8.65 (± 1.5)			
TCS	Triclosan	Antibiotics and Antimicrobials	51 (± 5.5)	67.9 (± 17)	38.4 (± 7.4)	47.8 (± 5.2)	–		197 (± 35)		> 253	> 253
AMP	Ampicillin	Antibiotics and Antimicrobials	NA	NA	NA	–	–		NA			
CBZ	Carbamazepine	Pharmaceuticals	NA	NA	NA	–	–		NA			
DBP	Dibutylphthalate	Industrial Chemicals	NA	NA	NA	–	–		NA			
DON	Deoxynivalenol	Mycotoxins	NA	NA	NA	–	–		NA			
ETU	Ethylene thiourea	Industrial Chemical	NA	NA	NA	–	–		NA			
IOP	Iopanoic acid	Pharmaceuticals	NA	NA	NA	–	–		NA			
PCL	Perchlorate	Industrial Chemicals	NA	NA	NA	–	–		NA			
SA	Salicylic acid	Personal Care Products	NA	NA	NA	–	–		NA			
T4	3,3′,5,5″-Tetraiodo-L-thyronine	Thyroid Hormone	NA	NA	NA	–	–		NA			
TPP	Triphenyl phosphate	Industrial Chemicals	NA	NA	NA	–	–		NA			

NA means not active up to the highest tested concentration specified in Methodology. “–” not tested

^a^IC_20_ is shown in case where IC_50_ was not reached

^b^*n* = 2

^c^*n* = 1 in ML-1 cells due to their very slow growth and comparative effective values to other cell lines, only a few compounds were screened in the ML-1 model

### Cell lines and culture conditions—methodology for cell culture of selected in vitro models

The Nthy-ori 3-1 (RRID: CVCL_2659), an immortalized human thyroid follicular epithelial cell line obtained from Sigma-Aldrich (Prague, Czech Republic), was cultured according to the supplier’s protocol in RPMI 1640 supplemented with 1% v/v Glutamax (GIBCO, Thermo Fisher Scientific, Prague, Czech Republic) and 10% v/v FBS (Biosera, BioTech, Prague, Czech Republic).

The FRTL-5 (RRID:CVCL_0265), a spontaneously immortalized rat thyroid follicular cell line obtained from Sigma-Aldrich, was cultured in Coon F12 medium (Sigma-Aldrich) supplemented with L-Glutamine, hydrocortisone, somatostatin, TSH and 10% FBS.

The ML-1 (RRID:CVCL_H525), a human thyroid gland follicular carcinoma cell line obtained from DSMZ-German Collection of Microorganisms and Cell Cultures (Braunschweig, Germany), was cultured according to the manufacturer’s protocol in DMEM media (Sigma-Aldrich) supplemented with 1% v/v Glutamax and 10% FBS.

HepG2 (RRID:CVCL_0027), a human hepatoma cell line was obtained from ATCC (LGC Standards, Lomianki, Poland) and cultivated in DMEM supplemented with non-essential amino acids (Sigma-Aldrich) and with 10% FBS.

The HeLa9903 (RRID:CVCL_2485), a human epithelial cervix carcinoma cell line was obtained from JCRB Cell bank (Osaka, Japan) and were cultivated in DMEM without phenol red (PAA, Austria) supplemented with 10% dextran-charcoal-treated FBS (Sigma-Aldrich).

The HEK293T (RRID:CVCL_0063), a human embryonal kidney SV40 transformed immortalized cell line obtained from ATCC, as well as the derived TPO-transfected HEK293T cell line, were cultured in DMEM High Glucose media (Biosera, BioTech) supplemented with 10% FBS.

### Production of stable human TPO-expressing HEK293T cell line

A human TPO (hTPO) sequence was obtained from NCBI (NM_000547.5) and it was used for the production of lentiviral vector by VectorBuilder (Malvern, USA) containing the gene of interest under the transcriptional control of CMV promotor and EGFP gene linked with puromycin resistance gene under control of EFS promotor (pLV[Exp]-CMV > {NM_000547_5}-EFS > EGFP:T2A:Puro). The purified vector was used to produce lentiviral particles in Lenti-X cell line (TakaraBio Inc., Göteborg, Sweden). The cells were exposed to equimolar mixture of plasmids (pMD2.G, Addgene #12259; psPAX2, Addgene #12260), GFP and NM_000547_5-containing plasmid together with polyethylenimine (PEI, 1 mg/mL; Polysciences) in a ratio of 2:1 (PEI:DNA) in OptiMEM (Sigma-Aldrich). The medium was replaced 3 h after exposure. The conditioned lentiviral medium was harvested 48 and 72 h later, frozen and stored at − 80 °C. HEK293T cells were exposed to the conditioned lentiviral medium supplemented with Polybrene (10 µg/mL, Merck, Prague, Czech Republic). After 24 h, the medium was replaced with fresh medium with puromycin (Invivogen, Toulouse, France) as a selection factor. After the puromycin treatment, cells were sorted on a FACS sorter FACSAria Fusion (BD Biosciences, Prague, Czech Republic) to obtain GFP-expressing cell clones that were further characterized.

After gene expression screening of single-cell clones and the mixed cultures by qPCR, the four single-cell lines with the highest TPO expression (HEK-TPOA7, HEK-TPOA2, HEK-TPOB12, and HEK-TPOB3) were selected for further characterization. The stability of TPO gene expression across different passages of HEK-SCA7 was assessed by qPCR.

### RNA isolation, gene expression analyses

The samples from the investigated cell lines were prepared following the protocol of the Qiagen RNeasy micro kit (Hilden, Germany). The concentration of the RNA sample was determined using the NanoDrop® 1000 spectrophotometer (ThermoFisher Scientific, Prague, Czech Republic). A total of 1000 ng RNA was transcribed into cDNA using the SensiFAST cDNA Synthesis Kit (LabMark, Prague, Czech Republic) in a PCR-thermocycler (Biometra, Jena Analytik, Germany) and subsequently tenfold diluted.

For the quantitative polymerase chain reaction (qPCR), the Kappa SYBR® Fast Universal kit (Merck) was used, and the reactions were conducted in a LightCycler® 480 instrument (Roche, Prague, Czech Republic) following the manufacturer’s protocol. The specific primer sets used for human TPO and rat TPO (Table S2) were designed using the NCBI Primer blast tool and obtained from Elisabeth Pharmacon (Brno, Czech Republic).

The data were analyzed in the LightCycler® 480 software using Absolute quantification (2nd derivative, 40 cycles LOQ) and Tm calling (Melt curve genotyping) methods. Each sample was analyzed in technical duplicates, and the housekeeping gene ACTB was used. Melting curves were generated and compared to positive control samples (total RNA Human Total Thyroid, HTT (Invitrogen, Thermo Fisher)); total RNA Human Total Liver, HTL (Invitrogen, Thermo Fisher) and total RNA Rat Total Thyroid, RTT (pooled from 400 male/female Sprague–Dawley rats, age: 8–12 weeks) (Takara Bio Europe).

### Western blot

In addition to gene expression profiling using qPCR, Western blotting was used to verify TPO protein expression level in different cell lines. Samples for western blotting were prepared using RIPA Lysis Buffer (50 mM Tris HCl, 150 mM NaCl, 0.5% sodium deoxycholate, 0.1% sodium dodecyl sulphate (SDS), 0.01% NaN_3_ and 1% Tween 20) with cOmplete™ Protease Inhibitor Cocktail (Roche) according to the manufacturer’s protocol.

The protein concentration was determined using the Bio-Rad DC-protein assay on SynergyMX fluorimeter (Biotek Agilent, Stevens Creek, USA). Subsequently, 10 µg of protein were loaded onto a 12.5% bis-acrylamide gel and subjected to SDS–Polyacrylamide gel electrophoresis (SDS-PAGE) using the Mini-PROTEAN system (Biorad, Prague, Czech Republic) at 150 V for 1.5 h. Following gel electrophoresis, the protein samples were blotted onto Immobilion-P PVDF membranes for 1 h and then blocked for an additional hour in 5% w/v skim milk. The membranes were subsequently incubated overnight with primary antibodies anti-hTPO (ab133322, Abcam, Cambridge, MA, USA; 1:2500) and anti-GAPDH (MAB374, Milipore, Prague, Czech Republic, 1:2000), followed by 1-h incubation with HRP-conjugated secondary antibodies anti-mouse (7076S, Cell Signalling Technology, Prague, Czech Republic, 1:2500) and anti-rabbit (7074S, Cell Signalling Technology, Prague, Czech Republic, 1:2500). Protein detection was accomplished using the GelDoc system (UviTec, Cambridge, UK) after applying the ECL chemiluminescent substrate (Clarity western ECL substrate, Bio-Rad, Prague, Czech Republic). Recombinant human TPO protein (hTPO standard; LS-G53963, LSBio, Seattle, USA) was used as a positive control, together with lysates from Hela and HepG2 as negative controls.

### TPO preparation

Cell lysates were derived from the cultured cell lines as the whole cell extract as described previously (Jomaa et al. 2015). For the human TPO-expressing HEK293T cell lines, cells were exposed to hematin (1 µg/mL) 2 days before the collection as described previously (Schmutzler et al. 2007). HEPES solution (Sigma-Aldrich) was added to balance the pH.

Briefly, the cells were washed with phosphate-buffered saline (PBS), scraped, resuspended in PBS, and centrifuged for 5 min at 150 rcf. The supernatant was discarded, the pellet was lysed with 0.1% m/v sodium deoxycholate (DC, in PBS) and incubated on ice for 20 min. Lysed cells were centrifuged for 5 min at 12,000 rcf to extract the soluble protein fraction. The supernatant protein concentration was measured using the Bio-Rad DC-protein assay following the manufacturer’s protocol. The cell lysates were kept at − 80 °C for longer-term storage.

### The TPO luminol assay

This assay has been carried out as described previously (Jomaa et al. 2015). It was performed in the white 96-well plates with total volume of reaction mixture 200 μL per well containing 100 μL cell lysate with protein content of 0.12 mg/mL (final 0.06 mg/mL), 100 μl GNE solution [1 M glycine–NaOH (pH 9.0), 1 mM EDTA] with the test chemical in MeOH (1% MeOH final concentration). The plate was incubated for 30 min with gentle shaking at 37 °C. The reaction was initiated by adding 20 μL of luminol solution using a dispenser (400 μM luminol in GNE solution; final conc. 35.6 μM) and 5 μL 80 mM H_2_O_2_ (final conc. 1.78 mM) following a 4-s delay by a dispenser. After 2 s delay, luminescence was measured in relative luminescence units (RLUs) in light emission at 428 nm integrated over 10 s using Synergy MX (Biotek Agilent, Stevens Creek, USA) plate reader with two dispensers.

### The AUR-TPO assay

This previously described assay (Paul et al. 2014) has been modified in the current study. Lower final volume and concentration of H_2_O_2_ were used. The assay was performed in the black 96-well plates with a final volume of 100 μL per well containing 50 μL cell lysate protein content of 0.12 mg/mL (final 0.06 mg/mL), and 20 µL PBS with 1 µL of the test chemical in MeOH (1% MeOH final concentration). The reaction was initiated by adding 25 μL of 100 μM (final conc. 25 μM) AUR solution in PBS and 5 µL of 800 μM H_2_O_2_ (final conc. 40 µM). After 30 min incubation at 37 °C in the dark, the fluorescence endpoint was measured on plate reader Synergy MX (Biotek Agilent, Stevens Creek, USA) at 544 nm excitation/590 nm emission. Nonspecific influence of the chemicals themselves on the fluorescence signal was assessed by measuring the fluorescence signal after co-incubating the chemicals (30 min at 37 °C) with 0.3 µM Resorufin and 40 µM H_2_O_2_ with the same excitation/emission spectra as with the AUR.

### Experimental strategy and optimization of assay conditions

In our study examining the protein concentration–response relationship within both the AUR and Lumi assays, we utilised varying final protein concentrations in the cell lysate. These concentrations were set at 0.015, 0.03, 0.06, 0.12, 0.18, and 0.6 µg/µL, with an exception for the ML-1 cell line, where they were 0.015, 0.03, 0.06, 0.12, 0.18, and 0.24 µg/µL. To ensure reliability, this experiment was conducted thrice, each time employing at least two different batches of lysates from each cell line.

Assessment of hematin pre-treatment impact: subsets of HEK-TPOA7, HEK293T, and FRTL-5 cells were pre-treated with hematin (1 µg/mL) two days prior to lysate collection. The cell lysates prepared from cells both with and without hematin pre-treatment, were subsequently employed in the protein concentration–response relationship studies within the AUR and Lumi assays to assess the impact of hematin addition into cultivation media on TPO activity.

Test of the effect of H_2_O_2_ concentration on the TPO activity measurement in the AUR assay: To assess the impact of H_2_O_2_ level on the AUR assay, a subset of chemicals (Table S6) was subjected to analysis at two H_2_O_2_ concentrations, 40 µM and 300 µM, to evaluate any variance in response.

Chemical testing strategy: In the assessment of the effects of selected chemicals, initially each chemical was tested at four concentrations (0.2; 2; 20; 200 μM; except of T3 and T4 with lower solubility—see below), with each concentration tested in triplicates. In case no activity was observed, the testing was repeated two more times to confirm the inactivity. In case the chemical was active in the initial test, it was further tested with six concentration dilution series based on the results of the first range-finding experiment in at least two more independent experiments. The final tested concentration ranges of chemicals were between 0.002 and 200 μM, except T3 (10, 5, 2.5, 1.25, 0.625, 0.3125 μM) and T4 (2, 1, 0.5, 0.25, 0.125, 0.0625 μM). For the detailed ranges of tested concentrations see the dose–response curves in Fig. 4.

### Data evaluation

For the data analysis, GraphPad Prism™ (GraphPad Software, San Diego, USA) was used for curve-fitting and IC_50_ or IC_20_ calculation. The signal (RLUs or fluorescence intensity) from the wells-containing cell lysate and chemical was background corrected (average RLUs or fluorescence intensity of lysate-free wells), then normalized to the solvent control (100% activity). Fold change of signal intensity was calculated by dividing the signal from cell lysate-containing wells by the signal from the background (average of cell lysate-free wells).

### Assessment of potential cross-species relevance

Sequence Alignment to Predict Across Species Susceptibility (SeqAPASS) web-based tool was used to assess the presence of TPO protein target for chemical interactions across species and the cross-species similarity of its amino acid sequences. Two levels of analyses were used. Level 1 compared complete primary amino acids of the proteins of interest amongst species using human TPO sequence AAA97517.1. In Level 2, the analysis focused specifically on the functional domains of the protein (Lalone et al. 2018) using sequence cd09825. In this analysis, *Rattus norvegicus, Mus musculus, Xenopus laevis, Cyprinus carpio*, *Oncorhynchus mykiss*, *Danio rerio*, and *Oryzias latipes* were chosen as model species to compare with *Homo sapiens*. The details on SeqAPASS can be obtained from the user guide (https://www.epa.gov/comptox-tools/seqapass-user-guide).

## Results and discussion

### Gene and protein expression

**Fig. 1:**
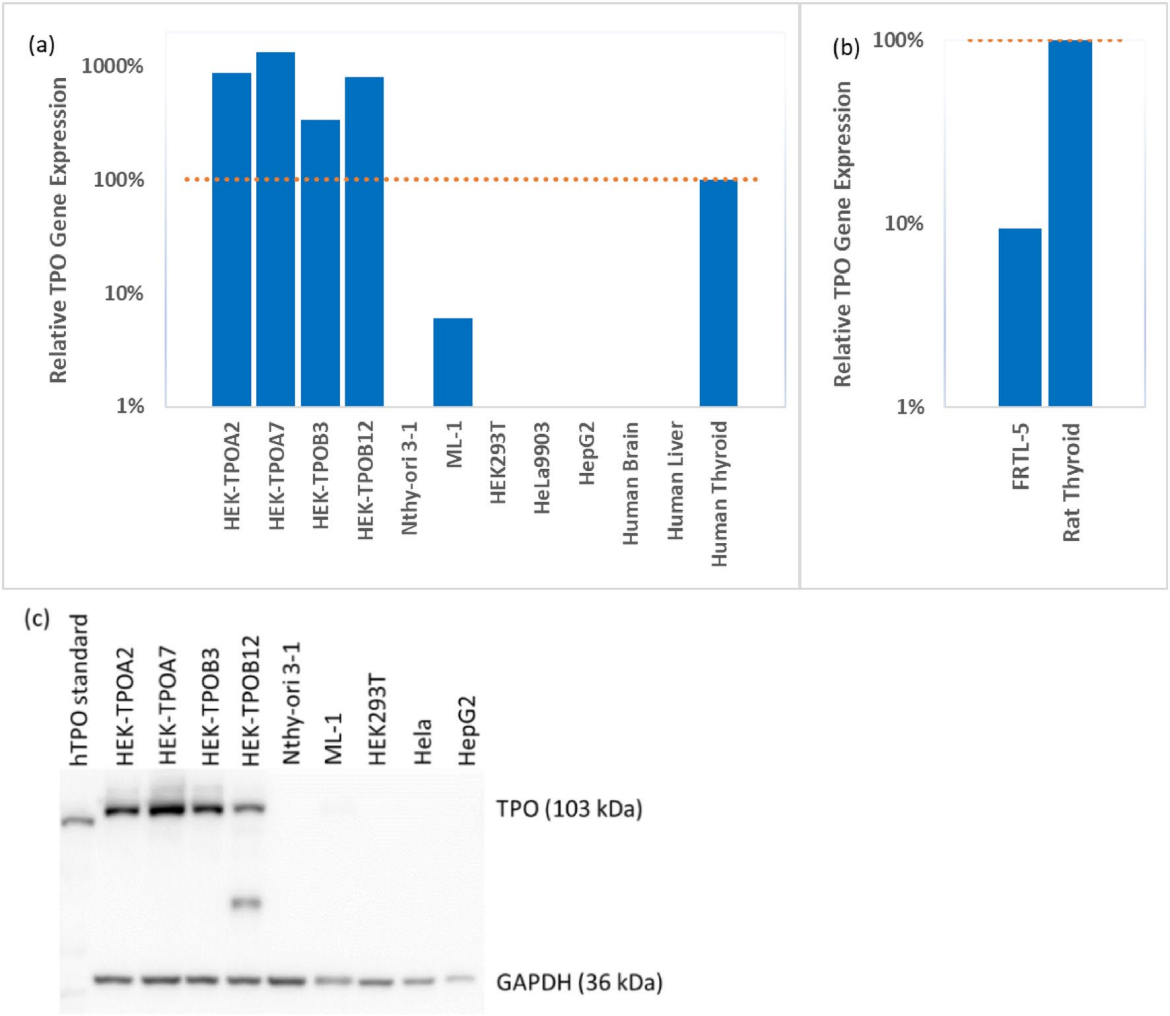
Comparison of the TPO gene and protein expression among groups of transfected cells, different in vitro models, and reference human (**a**) and rat (**b**) thyroid samples. Relative TPO Gene Expression values are related to TPO expression in the reference commercially available standards of total RNA from Human (**a** HTT) and Rat Total Thyroid (**b** RTT). **a** gene expression in human in vitro models (Nthy-ori 3-1, HEK293T, HeLa9903, HepG2, ML-1), human TPO-transfected cell lines (HEK-TPOA2, HEK-TPOA7, HEK-TPOB3, HEK-TPOB12) and reference samples from different tissues normalized to TPO expression in the human total thyroid; **b** gene expression in rat in vitro model (FRTL-5) compared to reference sample from rat thyroid. **c** TPO protein expression in the transfected cell lines and different human in vitro models. TPO band is around 103 kDa, and GAPDH is around 36 kDa. HEK-TPOA2, HEK-TPOA7, HEK-TPOB3, and HEK-TPOB12 are hTPO-transfected cell lines. TPO band cannot be found in Nthy-ori 3-1, ML-1, HEK293T, Hela, and HepG2 cell lines. Two bands were found in the HEK-TPOB12 sample (smaller TPO fragment). hTPO standard (842 amino acids) was from LSbio company. We have transfected the full length of TPO, which is 933 amino acids

Amongst the investigated stable cell lines from thyroid [Nthy-ori 3-1, ML-1 (human) and FRTL-5 (rat)] or other tissues [HEK293T (kidney), HeLa9903 (cervix), and HepG2 (liver)], the gene profiling of non-transfected cell lines unveiled detectable consistent endogenous TPO expression only in human ML-1 cells and rat FRTL-5 cells. Notably, the levels of TPO expression in these cell lines were approximately 17 times and 11 times lower compared to HTT and RTT (commercially available standards of total RNA from Human and Rat Total Thyroid), respectively (Fig. 1a, b).

TPO gene expression was much higher in all assessed single-cell line models of human TPO-transfected HEK cells, whilst it was not detectable in non-transfected HEK293T cells. After the optimization of the vector design, we obtained several TPO-expressing HEK293T clones, in which TPO expression was confirmed both on gene and protein level (Fig. 1). The TPO gene expression was highest in the transfected single-cell culture HEK-TPOA7, exhibiting an approximately 13-fold higher expression compared to the HTT. Also, HEK-TPOA2 and HEK-TPOB12 cell lines showed an approximately eightfold greater TPO expression compared to HTT, followed by HEK-TPOB3 displaying threefold greater expression than the reference HTT (Fig. 1). Furthermore, the TPO gene expression of HEK-TPOA7 was shown to be relatively stable across passages in cultivation (Fig. S1a in Supplementary Materials).

In the protein expression analysis, no TPO-specific band was detectable in either non-transfected HEK293T, HepG2 and HeLa9903 human non-thyroidal cell models or in the human thyroidal cell lines ML-1 and Nthy-ori 3-1 (Fig. 1c). In FRTL-5 cell line, two bands of TPO-antibody reactive proteins were observed, with the smaller size indicating possible TPO fragment (Fig. S2).

Observable TPO-specific bands were detected in all assessed TPO-transfected single-cell line models, including HEK-TPOA2, HEK-TPOA7, HEK-TPOB3, and HEK-TPOB12 at around 103 kDa. However, another truncated human TPO- protein band, appearing at approximately 57 kDa molecular weight, was also detected in HEK-TPOB12 (Fig. 1c). The most robust TPO protein as well as gene expression amongst the novel developed transfected models was observed in HEK-TPOA7 cell line, which was prioritised for further characterization of TPO activity and the development of the assay for TPO inhibition assessment.

ML-1 cells did not show detectable TPO protein expression in the WB analysis despite the detected TPO gene, possibly due to the low protein expression levels. Only FRTL-5 had detectable endogenous levels in both TPO gene and protein expression (Figs. 1b, S2). But the TPO gene expression levels in FRTL-5 was significantly lower than in commercially available rat thyroid reference sample (pooled from 400 male/female Sprague–Dawley rats, age: 8–12 week).

### Relationship of protein concentration with peroxidation activity in AUR and Lumi assay

**Fig. 2:**
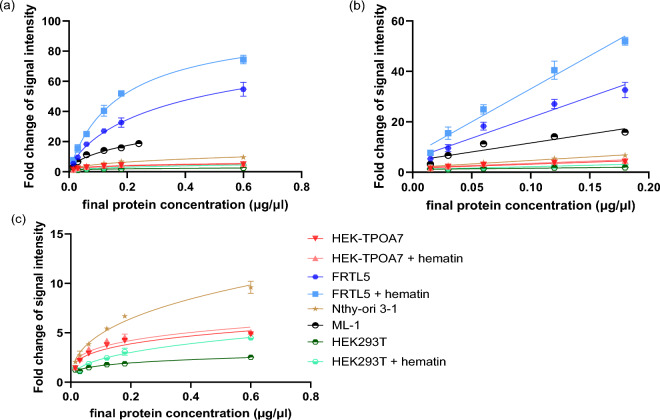
Protein concentration–response relationship of signal intensity fold change in TPO transfected cell line (HEK-TPOA7) and other in vitro models assessed by Luminol assay. Fold change of signal intensity was calculated by dividing the relative luminescence units (RLUs) from cell lysate-containing wells by the RLUs from the background (average of cell lysate-free wells). A fold change value close to 1 indicates minimal or undetectable peroxidation activity. For HEK-TPOA7, HEK293T, and FRTL-5, there were variants with/without treatment with hematin two days before the collection of cell lysates. **a** The protein concentration range of samples was 0.015–0.6 µg/µL except ML-1 model, where the highest concentration was 0.24 µg/µL. **b** subset of **a** chart containing a lower protein concentration range (0.015 to 0.18 µg/µL). **c** Lower fold change subset of **a** chart after excluding the FRTL-5 and ML-1 data

To demonstrate if cells can produce sufficient levels of catalytically active enzyme with activity detectable using AUR and Lumi assay, increasing concentrations of lysates were used in both these assays. In the Lumi assay, the luminescent signal was found to increase with protein concentration across all cell lines regardless of detectable TPO gene or protein expression, with FRTL-5 notably displaying the highest luminescence intensity at the same protein concentration compared to the other cell lines. In the lower concentration range (ranging from 0.015 to 0.18 µg/µL), all cell lines exhibited a nearly linear luminescence-protein relationship (*R*^2^ ≥ 0.80), whilst it was less linear at higher protein concentrations (Fig. 2). Importantly, the intensity of the luminol luminescence emitted by HEK-TPOA7 cell lysate with TPO overexpression was low and only slightly greater than that produced by non-transfected HEK293T cells with no TPO at the equivalent protein concentrations (Fig. 2).

The results demonstrate that the luminescence signal in the Lumi assay was not associated specifically with TPO activity, and it could be potentially attributed to the activity of other common peroxidases. One such example is Glutathione Peroxidase (GPx), which plays a pivotal role in safeguarding cells against oxidative damage by reducing hydrogen peroxide and lipid hydroperoxides (Pei et al. 2023). It seems that the Lumi assay can detect unspecific and broad peroxidation in the cellular homogenates of the studied models. However, further research would be needed to characterise the particular peroxidases playing a role in the detected activity across the different cell lines.

**Fig. 3:**
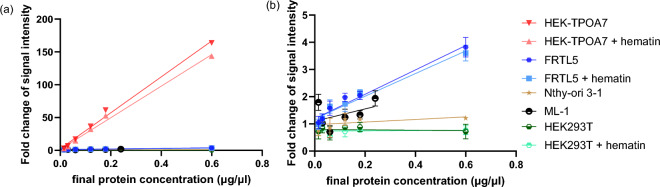
Protein concentration–response relationship of signal intensity fold change in TPO transfected cell line (HEK-TPOA7) and other in vitro models assessed by AUR assay. Fold change of the signal intensity was calculated by dividing the fluorescence intensity from cell lysate-containing wells by the fluorescence intensity from the background (average of cell lysate-free wells). A fold change value close to 1 indicates minimal or undetectable peroxidation activity. For HEK-TPOA7, HEK293T, and FRTL-5, there were variants with/out treatment with hematin 2 days before the collection of cell lysates. The final protein concentration of samples was 0.015–0.6 µg/µL except of the ML-1 model, where the highest concentration was 0.24 µg/µl. A strong increase of response with increasing protein content was found for the HEK-TPOA7 lysate. **b** the subset of **a** after the exclusion of the HEK-TPOA7 data

In the AUR assay, strongly increasing fluorescence signals with higher protein content were exclusively observed in HEK-TPOA7. The presence of hTPO in HEK-TPOA7 triggered a protein concentration-dependent linear increase in AUR fluorescence signal (*R*^2^ ≥ 0.98). FRTL-5 cells exhibited a similar concentration-dependent increase in fluorescence, though much lower TPO activity compared to HEK-TPOA7. ML-1 cells displayed an increase in fluorescence, albeit only at higher protein concentrations and to significantly lesser extent than in case of both HEK-TPOA7 and FRTL-5 lysates. Notably, neither HEK293T nor Nthy-ori 3-1, the cell models without detectable TPO expression, were able to produce elevated fluorescent signal even at high protein concentrations (Fig. 3).

The presented results demonstrate that the AUR assay is highly specific to the activity of TPO. This was evident from the increase in fluorescence with increasing protein levels observed in HEK-TPOA7 and FRTL-5 cells, which express TPO gene and protein, whilst the cell lines without TPO expression did not show any peroxidation in AUR assay (Fig. 3). Nevertheless, both FRTL-5 and ML-1 models showed only low fold change of fluorescence intensity reflecting very low TPO activity, which renders them unsuitable for sensitive HTS in the AUR assay. The high fold change of fluorescence intensity with increasing protein observed for HEK-TPOA7 lysate and stable TPO activity detected across relatively large span of passages (Figure S1b) document that the transfected hTPO gene is functional. Thus, this novel developed cell line can serve as an efficient source of TPO in the collected cell lysates.

Due to their limitations summarised in Table S3, namely poor growth characteristics, low TPO expression and the expensive media required, the tested stable cell lines (Nthy-ori, FRTL-5, ML-1) derived from thyroid were not suitable for routine HTS purposes. Therefore, transfected models overexpressing TPO were developed to provide enough functional TPO for high throughput testing.

For that purpose, the developed stably transfected HEK293T cells with approximately 30 h doubling time grow much faster than the thyroid-derived cell models and provide for fast protein production. Amongst all assessed cell lines, HEK-TPOA7 exhibits the most robust and consistent TPO gene and protein expression and demonstrates rapid growth (Table S3). This implies its capability to consistently and efficiently supply a substantial amount of TPO protein for the assessment of its inhibition.

### Hematin treatment influence

Whilst cultivating the HEK-TPOA7, HEK293T and FRTL-5, the potential impact of hematin addition on peroxidation activity was tested by comparing variants with or without treatment with hematin 2 days before the collection of cell lysates. It has been reported in the literature that haem group is an important cofactor essential for the enzymatic activity of TPO (Ruf and Carayon 2006). Thus, we tried to assess if hematin addition would impact the enzyme activity by running both Lumi and AUR assays with and without hematin addition in concentration described in literature (Schmutzler et al. 2007).

In the AUR assay, the fluorescence signals were comparable between the hematin-treated and non-treated samples (Fig. 3). Moreover, there was no significant difference in IC_50_ values of the tested compounds between the lysates prepared from cells cultivated with or without hematin (Table S4). This indicates that hematin’s addition is not necessary for the TPO AUR assay for the cell lines cultivated under the conditions used in our study.

However, the Lumi assay indicated a significant increase in signal for FRTL-5 and HEK293T samples after adding hematin (Fig. 2). Although the hematin treatment increased the luminescence signal, it did not have much impact on IC_50_ values of the model compounds (Table S4). The observed enhancement of signal in the Lumi assay in lysates from cell lines both with and without detectable TPO expression, attributed to hematin, may indicate that hematin had an influence on non-specific peroxidase activity in the cells.

### H_2_O_2_ influence in the AUR assay

In comparison to the studies by Paul et al. (2014) and Friedman et al. (2016) (300 µM final concentration), we utilised a lower concentration of H_2_O_2_ (40 µM final concentration) in the AUR assay due to some artefacts connected with high H_2_O_2_ levels in parallel experiments (part of a follow-up study, data not shown here). The results demonstrate that the fold change of fluorescence signal intensity reflecting the TPO activity between these two concentrations of H_2_O_2_ in AUR assay was comparable or slightly greater at 40 µM (Table S5). When we compared the IC_50_ values for various chemicals across these two H_2_O_2_ concentrations, we found that there were no significant differences. This suggests that both H_2_O_2_ concentration are sufficient for the functionality of the assay with comparable outcomes of IC_50_ determination for the tested chemicals (Table S6).

### Peroxidase activity inhibition by chemical exposure

**Fig. 4:**
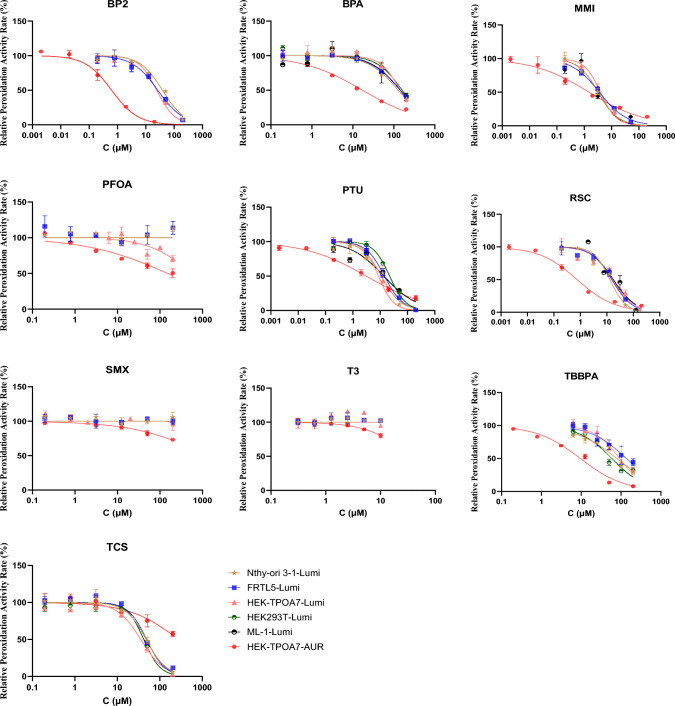
Comparison of dose–response curves for positive chemicals among lysates from different cells and assays. Relative peroxidation activity rate (%): The RLUs or fluorescence intensity from the well-containing cell lysate and chemical was background-corrected (average RLUs or fluorescence intensity of lysate-free wells), then normalized to the solvent control (100% activity)

Figure 4 and Table 1 display the dose–response curves and IC_50_ or IC_20_ values and for all tested chemicals, which were obtained from either Lumi assay or AUR assay.

In the applied Luminol assay, bisphenol A (BPA), 6-propylthiouracil (PTU), triclosan (TCS), 2,2′-4,4′-tetrahydroxy benzophenone (BP2), resorcinol (RCS), and methimazole (MMI) showed luminescence inhibition across Nthy-ori 3-1, FRTL-5, and HEK-TPOA7 cell lines, allowing for quantification of IC_50_ concentrations that were comparable across the cell models, except for PFOA, which allowed for quantification of IC_20_ only with HEK-TPOA7 cell lysate (Table 1).

On the other hand, in the AUR assay, BPA, PTU, TBBPA, TCS, BP2, RSC, and MMI showed strong fluorescence inhibition potency, enabling quantification of IC_50_ values, whilst PFOA, T3, and SMX exhibited relatively lower inhibition, which enabled to derive only IC_20_ values in the HEK-TPOA7 cell line (Table 1). There was no significant nonspecific impact of the chemicals on the fluorescence in the AUR assay, except of a minor influence in the case of triclosan, which could be related with the presence of precipitate in the exposure variant without the cell lysate (Table S7 and Fig. S3).

As is obvious from Fig. 4, the dose-–response curves in the Lumi assays were similar for all chemicals across all the tested cell lines regardless of the detectable presence of TPO gene and protein. On the other hand, the AUR assay enabled the measurement of TPO inhibition only in the case of HEK-TPOA7 cell line with high TPO expression. As written above, the other cell lines showed either rather low (FRTL-5; ML-1) or undetectable TPO activity in AUR assay, which corresponds to their level of TPO gene and protein expression.

Interestingly, although as our data show, the Lumi assay is not TPO-specific and probably reflects the general peroxidase activity, it identified the same inhibitors as the TPO-specific AUR assay in our chemical set, though with higher effective concentrations (IC_50_ levels). There was a complete agreement between Lumi and AUR assays in the identification of nonactive and active inhibitory chemicals across all chemicals tested, with the exception of sulfamethoxazole (SMX). Nonetheless, the IC_50_ value for SMX in the AUR assay was relatively high (103 ± 14 µM). From the IC_50_ values presented in Table 1, it is evident that the AUR assay was more sensitive across most compounds. As the effective concentrations in AUR assay on HEK-TPOA7 cells were often more than order of magnitude lower than detected by Lumi assay across the different cell lines, it is not surprising that the low inhibitory effect of SMX was not detected in the Lumi assay. In general, these results suggest that even TPO non-specific assays reflecting general peroxidase activity might provide an indication of potential effect of chemicals on TPO activity or for prioritisation of chemicals for screening with more specific assays.

The AUR assay demonstrated greater specificity to TPO inhibition, but also greater sensitivity demonstrated by lower IC_50_ values compared to the Lumi assay in all cases with the exception of triclosan (TCS). The higher IC_50_ observed for TCS in the AUR assay for HEK-TPOA7 results could be attributed to differences in its solubility. Triclosan exhibits a solubility of 10 mg/L (equivalent to 34.5 µM) at 20 °C according to PubChem. Notably, the Lumi assay maintains a higher pH level 8.8 [100 µL total reaction volume contained 50 µL protein dilution (pH = 7.2) and 50 µL GNE buffer (pH = 9)], compared to pH 7.2 in the AUR assay, enhancing the solubility of TCS within this specific assay environment, as TCS is readily soluble in alkaline solutions (Dann and Hontela 2011; Lee et al. 2019). To assess the impact of pH, as the AUR assay for HEK-TPOA7 is conducted at normal pH 7.2, TCS effect was also examined by AUR assay under higher pH conditions (pH 8.8) corresponding to the Lumi assay. The resulting IC_50_ value for TCS in the AUR assay at higher pH was 72.7 µM (SD = 14), significantly lower than its IC_50_ value at standard pH 7.2 used in the AUR assay (Fig. S4). However, in the higher pH setting of the AUR assay for HEK-TPOA7, the fold change in signal intensity decreased to the range of 2.7–4.5, compared to more than 10 in the normal pH. These findings underscore the significance of considering the influence of pH on TCS solubility and its potential repercussion on assay outcomes.

We have compared our data to those reported in previous studies for different cell lines using Lumi or AUR assays (Table 1). Nevertheless, for most tested chemicals, including some widespread environmental pollutants, our study brought the first information on their potential to inhibit TPO activity using in vitro human cell-based detection system. In case of Lumi assay, there is only one corresponding study available (Jomaa et al. 2015) and our IC_50_ levels are in line with it. In the AUR assay, for the few previously tested compounds, IC_50_ levels of positive chemicals were comparable or in some cases little higher than in studies with other TPO-expressing cell models (Table 1) (Dong et al. 2020; Jomaa et al. 2015). There are various factors that may influence these differences, including the utilization of different TPO sequences in TPO overexpressing models. Notably, Dong et al. (2020) employed the BC095448 sequence, whereas our study utilised newer sequence NM_000547. Whilst they do not differ significanly in the translated region, the newer sequence has significanly longer untranslated region after the terminal codon which might affect the activity of the translated protein. In addition, variations in enzyme conformation and post-translational modifications across different cell lines, such as the use of CHO and LentiX in Dong et al. (2020) versus HEK293T in our transfection, could contribute to these differences. Anyway, our data show good level of agreement with similar studies and in vitro models, documenting the suitability of our HEK-TPOA7 cell model for TPO inhibition screening in combination with the AUR assay since it is TPO-specific and sensitive.

The currently established AUR assay in our and other studies specifically focuses on the peroxidation activity of the TPO. The assessment of TPO’s ability to iodinate and link iodothyronines through ether bonds needs further development of methodology in the future studies.

It must be noted that the AUR data in Jomaa et al. (2015) are based on Nthy-ori 3-1 cells, for which our study did not detect measurable TPO gene or protein expression. Some previous studies (Godlewska et al. 2018; Tuncel et al. 2007; Ząbczyńska et al. 2020) reported TPO expression in this cell line, albeit at very low levels. Specifically, Tuncel et al. (2007) reported a very low TPO expression that would be below the limit of detection in our study (Ct of 35.5). These studies indicate that low TPO expression can be found in Nthy-ori 3-1 in some cases. This may be a reason why Nthy-ori 3-1 worked in the AUR assay in the study by Jomaa et al. (2015).

In addition, the Toxcast (Friedman et al. 2016) employed the AUR assay to identify TPO inhibitors, utilizing microsomes from 120 pooled rat thyroids. Despite the different species and sources of TPO (ex vivo thyroid tissue in ToxCast, in vitro TPO overexpressing cell line in our study), there is a good agreement in the identification of the TPO inhibiting and nonactive chemicals for 12 compounds that were tested both in ToxCast and our study. ETU was the only chemical that showed quantifiable IC_50_ in the rat thyroid-based assay in ToxCast, but not in our study.

The US EPA CompTox Chemicals Dashboard, referenced in Table S1, provides predictive data on human exposure to the listed chemicals. It includes estimated human exposure prediction data (Table S1) for all effective inhibitors detected in our study. Amongst the tested compounds, the top five chemicals with the highest predicted human exposure rates—Triclosan, Bisphenol A, Resorcinol, Iopanoic acid and Methimazole, cause varying degrees of TPO inhibition. Notably, Resorcinol and Methimazole show strong TPO inhibitory activity with IC_50_ values at 0.358 (± 0.16) µM and 1.50 (± 0.39) µM in the AUR assay. BPA is also an inhibitor of TPO, though at greater concentration. TCS, although the most prevalent amongst these chemicals, has a relatively low impact on TPO activity, affected also by solubility issues described above. On the other hand, amongst the strongest detected TPO inhibitors belongs commonly occurring benzophenone with relatively high predicted exposure rates and frequent presence in various aquatic ecosystems, as it is often utilised in sunscreens as a UV filter. These results document the ability of chemicals from different use groups, with diverse structures, including some widespread environmental pollutants to inhibit the activity of this critical enzyme controlling the TH synthesis. This highlights the importance and relevance of this mode of action and the need of TD considerations in the hazard and risk assessment.

### Cross-species relevance—SeqAPASS analysis

Extrapolation of toxicity data from model species to other species of concern is a considerable challenge in chemical hazard assessment. Differences in chemical sensitivities amongst species can range from a few to more than a thousand times (Doering et al. 2018). TPO inhibition has been connected with the adverse health effects across vertebrates including mammals, birds, amphibians, and fish (Noyes et al. 2019). Based on currently available Adverse Outcome Pathways (in different stages of development), TPO inhibition was identified as highly relevant molecular-initiating event for various vertebrate species linked with diverse adverse outcomes such as impaired neurodevelopment in mammals, swim bladder and eye development in fish and metamorphosis in amphibians (Holbech et al. 2020; Society for Advancement of AOPs 2024). In this work, an in silico tool developed by the US Environmental Protection Agency (EPA) Sequence Alignment to Predict Across Species Susceptibility (SeqAPASS) was used to predict the similarity of sensitivity to chemicals across animal species based on the protein structure (US EPA 2018). The amino acid sequence of the protein in a reference species, human in our case, was compared with the same protein sequence of other species. The higher is the similarity of the proteins of interest, the higher the probability the proteins will be susceptible to the same chemicals and thus cross-species relevance (LaLone et al. 2016). The Level 1 and level 2 analysis of SeqAPASS showed that human TPO is similar to corresponding proteins from other vertebrates, including the commonly used model mammalian, fish and amphibian species (Rat, Mouse, African clawed frog, Rainbow trout, Eurasian carp, Japanese rice fish and Zebrafish) (Table 2, Fig. S5). Furthermore, in the Level 2 functional sequence analysis, it was observed that the similarity scores for these model animal species consistently exceeded 50%. It means that data obtained from studies involving human TPO could inform research and assessment involving other vertebrate species, and vice versa, thereby facilitating cross-species insights and enhancing the translational relevance of experimental findings. The high level of cross-species similarity in susceptibility to TPO inhibition has been shown previously in pig and rat (Paul et al. 2013). The SeqAPASS analyses supports this finding and also demonstrates that the primary structure and functional domain of TPO show high level of similarity not only in mammals but also across other vertebrate classes as well (Table 2, Fig. S5; Haigis et al. 2023). It is likely that the results obtained from our hTPO transfected cell line can be applied qualitatively and indicate relevant TPO inhibitors also for other vertebrate species.

**Table 2:** SeqAPASS summary analysis report for TPO protein shows significant similarity across candidate animal model species when comparing whole protein (level 1) and the active domain (level 2)

Protein	Thyroid peroxidase (TPO)	
Reference species	Human	
Tested species	Level 1	Level 2
*Homo sapiens*	100%	100%
*Rattus norvegicus*	75.5%	82.1%
*Mus musculus*	74.6%	81.2%
*Xenopus laevis*	50.5%	66.1%
*Oncorhynchus mykiss*	40.8%	60.1%
*Cyprinus carpio*	40.1%	60.6%
*Oryzias latipes*	39.2%	55.9%
*Danio rerio*	37.7%	56.6%

As TPO inhibition has been linked through AOP network to adverse effects including impaired development across species, it is essential to characterise the TPO inhibiting potential of environmental exposure chemicals and their mixtures. The assessment of TPO inhibition by the environmental samples has been scarce so far, namely due to the lack of available high throughput in vitro based methods without the need for ex vivo materials. To our knowledge there is only a single study showing detection of TPO inhibition in rat thyroid microsomes by both surface and treated waste-water samples (Leusch et al. 2018). It is noteworthy that the effect was detected at environmental relevant concentration at least in case of the treated waste-water sample. This indicates that this endpoint could be significant for aquatic organisms exposed to environmental pollutant mixtures. This is supported by a wide range of studies that detected the chemicals that were identified as TPO inhibitors using ex vivo rat thyroid microsomes (Friedman et al. 2016) in different environmental matrices. These include compounds from diverse chemical groups such as polycyclic aromatic compounds, bisphenols, and current use pesticides in matrices such as surface waters (Novák et al. 2018; Sauer et al. 2023), house dust (Nováková et al. 2022; Pinto-Vidal et al. 2024), indoor air (Nováková et al. 2022), and ambient air (Nováková et al. 2020). Even though their detected concentrations can be relatively low, they are mostly part of complex mixtures with many other compounds for which TPO inhibition is unknown. This highlights the necessity of applications of the high throughput methods for the assessment of both individual chemicals, as well as relevant exposure mixtures.

## Conclusion

In conclusion, the AUR assay stands out as the specific method applicable for high throughput assessment of TPO activity inhibition in samples from cell line*s* with sufficient TPO activity, whilst the results of Luminol assay are not specific to TPO. We have successfully created a cell line transfected with human TPO, which efficiently produces human TPO that can be used for the assessment of the inhibition of its activity by chemicals, mixtures or environmental samples. This cell line can be utilised in HTS assays based on AUR, enabling the effective characterization of TPO inhibitors. Conducting screenings with this cell line generates data applicable for understanding the impact of TH-disrupting chemicals on the activity of this crucial enzyme in TH synthesis. The observation that chemicals from different use groups with diverse structures, including some widespread environmental pollutants, exhibit TPO inhibition ability, as well as its cross-species conservation and relevance for various adverse effects, underscore the need for efficient tools and approaches for the assessment of this mode of action. The results highlight the importance of TD considerations in the hazard and risk assessment, which is crucial for gaining a deeper understanding and implementing effective mitigation strategies for the environmental and health impacts of exposure chemicals and their mixtures.

### Supplementary Information

Below is the link to the electronic supplementary material.

Supplementary file1 (DOCX 1085 KB)

## Data Availability

Data can be made available upon request.
