# Supplementary material for: In vitro assessment of thyroid peroxidase inhibition by chemical exposure: comparison of cell models and detection methods
Source: Arch Toxicol. 2024 May 25;98(8):2631–45. doi: 10.1007/s00204-024-03766-7 (PMC11272733; doi:10.1007/s00204-024-03766-7)
Supplement: Supplementary file 1 — Supplementary file1 (DOCX 1085 KB) [file 204_2024_3766_MOESM1_ESM.docx]

**Supplementary Materials** to the paper:

## ***In vitro* assessment of thyroid peroxidase inhibition by chemical exposure – comparison of cell models and detection methods**

## Runze Liu, Jiří Novák, Klára Hilscherová*

## RECETOX, Faculty of Science, Masaryk University, Kotlarska 2, 611 37 Brno, the Czech Republic

## *Corresponding author: [klara.hilscherova@recetox.muni.cz](mailto:klara.hilscherova@recetox.muni.cz)

Table S1. The chemical name, abbreviation, CAS no., and source for all tested chemicals. Exposure Prediction data (µg/kg/day) for humans is from the US EPA CompTox Chemicals Dashboard - Demographics Predictions Data (<https://comptox.epa.gov/dashboard/>).

| **Abbrev.** | **Chemical** | **CAS No.** | **Exposure Prediction (µg/kg/day)** | **Catalog Number**  (Sigma-Aldrich) | **Structure** |
| --- | --- | --- | --- | --- | --- |
| AMP | Ampicillin | 69-53-4 | 1.32E-07 | 59349-100MG | 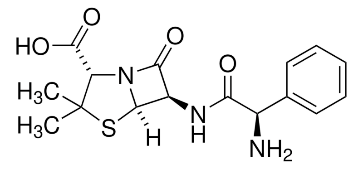 |
| BP2 | 2,2′-4,4′-tetrahydroxy benzophenone | 131-55-5 | 1.13E-03 | T16403-25G | 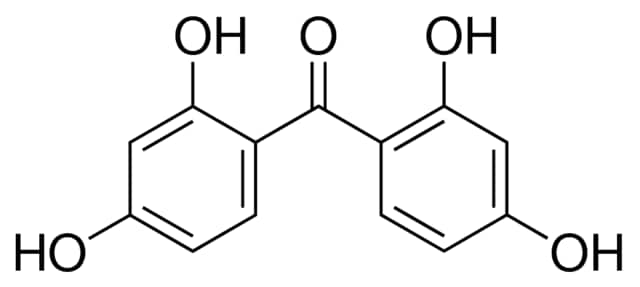 |
| BPA | Bisphenol A | 80-05-7 | 2.97E-02 | 239658-50G | 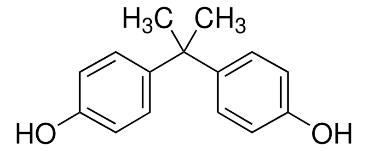 |
| CBZ | Carbamazepine | 298-46-4 | 3.27E-04 | 94496-100MG | 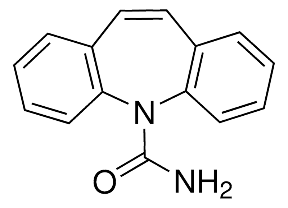 |
| DBP | Dibutylphthalate | 84-74-2 | 6.53E-04 | 524980-100ML | 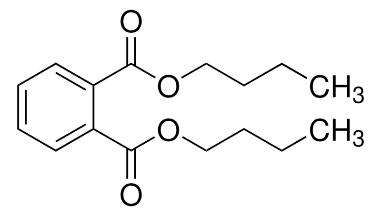 |
| DON | Deoxynivalenol | 51481-10-8 | 3.97E-04 | D0156-5MG | 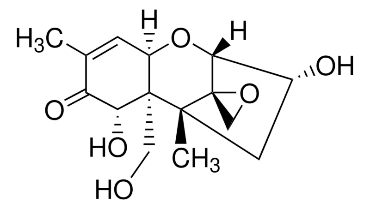 |
| ETU | Ethylene thiourea | 96-45-7 | 1.89E-05 | 45531-250MG | 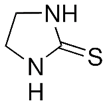 |
| IOP | Iopanoic acid | 96-83-3 | 3.07E-03 | 14131-100MG | 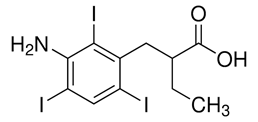 |
| MMI | Methimazole | 60-56-0 | 2.59E-03 | M8506-25G | 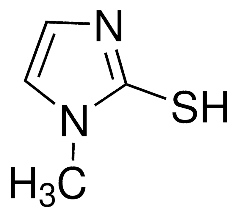 |
| PCL | Perchlorate | 7601-89-0 | No data | 410241-100G | 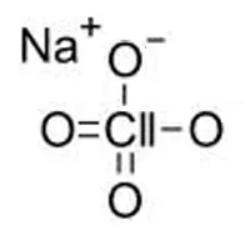 |
| PFOA | Perfluorooctanoic acid | 335-67-1 | 5.47E-05 | 33824-100MG | 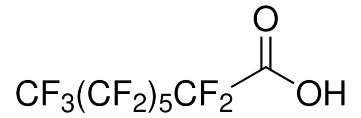 |
| PFOS | Perfluorooctane sulfonate | 2795-39-3 | 2.96E-10 | 77282-10G | 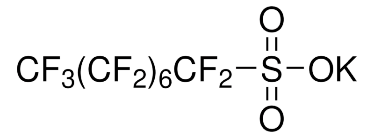 |
| PTU | 6-propylthiouracil | 51-52-5 | 5.05E-04 | P3755-25G | 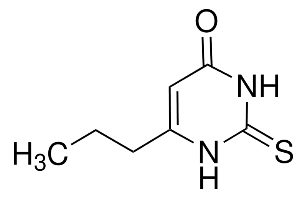 |
| RSC | Resorcinol | 108-46-3 | 1.91E-02 | 307521-100G | 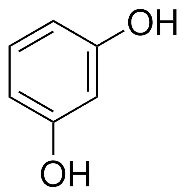 |
| SA | Salicylic acid | 69-72-7 | 2.41E-03 | S5922-100G | 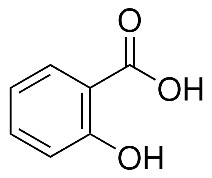 |
| SMX | Sulfamethoxazol | 723-46-6 | 2.64E-04 | S7507-10G | 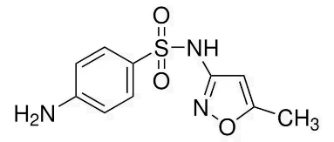 |
| T3 | 3,3′,5-Triiodo-L-thyronine | 55-06-1 | 1.47E-04 | T6397-250MG | 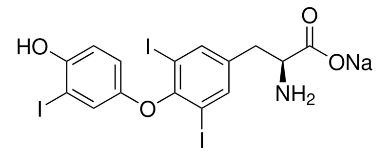 |
| T4 | 3,3′,5,5″-Tetraiodo-L-thyronine | 51-48-9 | 2.00E-04 | T2376-500MG | 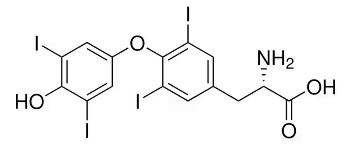 |
| TBBPA | Tetrabromobisphenol A | 79-94-7 | 1.28E-04 | 330396-100G | 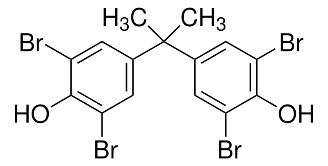 |
| TCS | Triclosan | 3380-34-5 | 1.97E-01 | PHR1338-1G | 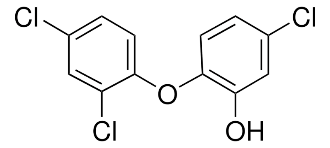 |
| TPP | Triphenyl phosphate | 115-86-6 | 2.30E-04 | 241288-50G | 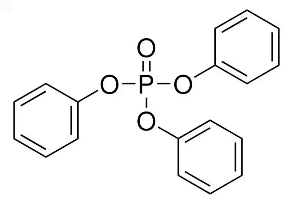 |

Table S2. Primer list used in the qPCR analyses of gene expression. FP = Forward primer; RP = Reverse primer

| Gene name | Primer | primer sequence |
| --- | --- | --- |
| human TPO | FP | GTCTGTCACGCTGGTTATGG |
|  | RP | CAATCACTCCGCTTGTTGGC |
| rat TPO | FP | GGGCATTGCACCAGATCATCA |
|  | RP | TGTAGCCTGGTAAGCCGTGG |
| ACTB | FP | CACCATTGGCAATGAGCGGTTC |
|  | RP | AGGTCTTTGCGGATGTCCACGT |


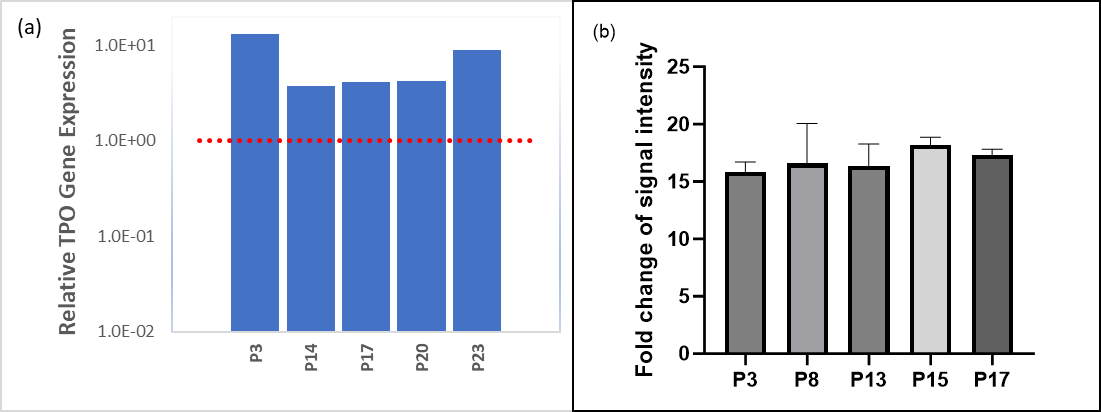


Figure S1. TPO gene expression and Fold change of the signal intensity in the AUR assay reflecting the TPO activity across different passages of HEK-SCA7. P means passage number. (a) TPO gene expression across different passages of HEK-SCA7 cells. Relative TPO Gene Expression values are related to TPO expression in the reference human thyroid sample (HTT) indicated by the red line. (b) Fold change of the signal intensity was calculated by dividing the fluorescence intensity from cell lysate-containing wells by the fluorescence intensity from the background (average of cell lysate-free wells) in the AUR assay.


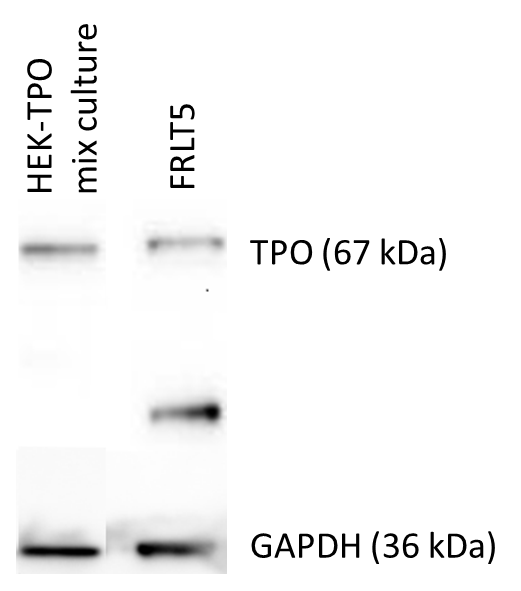


Figure S2. Protein Expression of FRTL-5 and HEK-TPO mix culture. TPO band is around 67 kDa, and GAPDH is around 36 kDa. An anti-TPO (N-terminal) antibody (SAB2109049), which can react with both rat and human TPO proteins, has been used. The HEK-TPO mix culture sample is prepared from the mixed culture of the developed human TPO-transfected cell lines.

Table S3. The summary of cell line characteristics. The values in TPO gene expression column show the Relative TPO Gene Expression compared to TPO expression in the reference commercially available standards of total RNA from thyroid. The results from Nthy-ori 3-1, ML-1, HEK-TPOA7, and HEK293T were normalized to TPO expression in human thyroid standard (HTT). The result from FRTL-5 was normalized to TPO expression in rat thyroid standard (RTT). TPO protein expression column shows the result of western blot analysis. The value is the grayscale intensity of the TPO band normalized to the GAPDH band. nd = nondetectable

| Cell line | Species | Tissue of origin | Doubling time | TPO gene expression | TPO protein expression |
| --- | --- | --- | --- | --- | --- |
| Nthy-ori 3-1 | human | thyroid-follicular epithelia | 36-48 hours | nd | nd |
| ML-1 | human | thyroid-follicular epithelia carcinoma | 4-7 days | 0.06 | nd |
| HEK-TPOA7 | human | hTPO transfected embryonic kidney | 30 hours | 13.2 | Strong band (6.57) |
| FRTL-5 | rat | thyroid-follicular epithelia | 5-7 day | 0.002 | Two bands (0.23) |
| HEK293T | human | embryonic kidney | 30 hours | nd | nd |

Table S4. IC_50_ values of chemicals in compared assays using lyzates of cells with (+) or without (-) hematin pre-treatment. There is no significant difference in IC_50_ between lyzates prepared from cells treated or untreated with Hematin. NT means not tested.

| Abbrev. | FRTL-5 | | HEK293T | | HEK-TPOA7 | | | |
| --- | --- | --- | --- | --- | --- | --- | --- | --- |
| Method | Lumi assay | | Lumi assay | | Lumi assay | | AUR assay | |
| Hematin | - | + | - | + | - | + | - | + |
| BPA | 143 (±17) | 166 (±22) | 122 (±32) | NT | 107 (±16) | 130 (±17) | 8.29 (±1.3) | 10.6 (±5.8) |
| PTU | 13.2 (±1.0) | 12.5 | 18.9 (±4.6) | NT | 18.4 (±1.2) | 8.53 (±1.1) | 3.52 (±0.68) | 5.36 (±0.9) |
| TBBPA | 130 (±6.9) | 190 (±10) | 64.4 (±6.7) | 87.1 (±3.4) | 107 (±11) | 90.9 (±20) | 8.2 | 8.65 (±1.5) |
| TCS | 67.9 (±17) | 46.2 | 47.8 (±5.2) | NT | NT | 38.4 (±7.4) | NT | 197 (±35) |
| BP2 | 29.6 (±13) | 31.4 | NT | NT | NT | 20.2 (±5.09) | 0.48 (±0.12) | 0.56 (±0.06) |
| RSC | 13.8 (±1.9) | 13.9 | NT | NT | NT | 17.9 (±1.0) | 0.36 (±0.17) | 0.36 (±0.16) |
| MMI | 3.91 (±0.67) | 3.59 (±0.28) | NT | NT | NT | 3.24 (±0.63) | 1.12 (±0.53) | 1.50 (±0.39) |

Table S5. Fold change of fluorescence signal intensity between different concentrations of H_2_O_2_ in AUR assay using different concentrations of HEK-TPOA7 protein. Fold change of fluorescence signal intensity was calculated by dividing the fluorescence intensity from cell lysate-containing wells by the fluorescence intensity from the background (average of cell lysate-free wells).

| H_2_O_2_ concentration | final protein concentration 0.6 mg/ml | final protein concentration 0.06 mg/ml |
| --- | --- | --- |
| 40uM | 192.5 | 15.8 |
| 300uM | 187 | 12.3 |

Table S6. IC_50_ values of chemicals in the AUR assay using different concentrations of H_2_O_2_ and HEK-TPOA7 lyzate (IC_20_ in case of SMX). There is no significant difference or general trend in IC_50_ values between the assays with the two H_2_O_2_ concentrations (n=2)**.**

| Abbrev. | Chemical | H_2_O_2_ 40uM | H_2_O_2_ 300uM |
| --- | --- | --- | --- |
| BPA | 2,2-Bis(4-hydroxyphenyl)propane (Bisphenol A) | 10.6 (±5.8) | 10.9 (±6.3) |
| TBBPA | Tetrabromobisphenol A | 8.65 (±1.5) | 6.5 (±1.2) |
| TCS | Triclosan | 197 (±35) | 165 (±24) |
| BP2 | 2,2′-4,4′-tetrahydroxy benzophenone | 0.56 (±0.06) | 0.64 (±0.03) |
| RSC | Resorcinol | 0.36 (±0.16) | 0.61 (±0.33) |
| MMI | Methimazole | 1.5 (±0.39) | 1.33 (±0.52) |
| SMX* | Sulfamethoxazol | 101 (±14) | 140 (±10) |
| T3* | 3,3′,5-Triiodo-L-thyronine | 11.1 (±1.2) | 10.5 (±1.0) |

Table S7. The results of the assessment of nonspecific influence of the chemicals themselves on the fluorescence signal. It was assessed by examining any potential increase in fluorescence or quenching after incubating the chemicals (30 min at 37°C) with 0.3 µM resorufin and 40 µM H_2_O_2_ and expressed as Relative Fluorescence Rate (%) compared to fluorescent standard resorufin. The fluorescence endpoint was measured on microplate reader Synergy MX (Biotek Agilent, Stevens Creek, USA) at 544 nm excitation/590 nm emission. Relative Fluorescence Rate (RFR; %) was calculated by dividing the mean fluorescence intensity of the wells with chemicals by the mean fluorescence intensity from the wells with solvent control (1% MeOH). Each chemical was tested in triplicate at the two highest tested concentrations used in TPO inhibition assay. There were mostly no effects, only PFOS, TCS, and TBBPA were found to cause slight signal inhibition/quenching (less than 20%).

|  | Mean RFR | SD | Mean RFR | SD |
| --- | --- | --- | --- | --- |
| Tested concentration | 100 uM |  | 200 uM |  |
| TCS | 84% | 6.2 | 83% | 2.6 |
| TBBPA | 94% | 9.2 | 83% | 4.3 |
| PFOS | 89% | 3.0 | 88% | 3.5 |
| AMP | 100% | 11.5 | 103% | 0.9 |
| BP2 | 101% | 5.1 | 106% | 2.2 |
| BPA | 103% | 5.4 | 104% | 4.6 |
| CBZ | 106% | 5.1 | 104% | 8.0 |
| DBP | 93% | 0.3 | 96% | 5.4 |
| DON | 103% | 1.2 | 109% | 8.1 |
| ETU | 109% | 4.2 | 110% | 3.0 |
| IOP | 103% | 2.1 | 97% | 7.4 |
| MMI | 106% | 1.4 | 104% | 6.6 |
| PCL | 108% | 7.5 | 106% | 6.8 |
| PFOA | 97% | 9.6 | 95% | 2.5 |
| PTU | 96% | 4.2 | 98% | 4.7 |
| RSC | 98% | 2.5 | 99% | 6.3 |
| SA | 102% | 3.8 | 101% | 6.6 |
| SMX | 109% | 8.5 | 109% | 1.8 |
| TPP | 93% | 7.3 | 92% | 2.9 |
| Tested concentration | 5 uM | SD | 10 uM | SD |
| T3 | 95% | 4.5 | 100% | 5.9 |
| Tested concentration | 1 uM | SD | 2 uM | SD |
| T4 | 103% | 6.1 | 99% | 6.5 |

Figure S3. The comparison of Relative Fluorescence Rate (RFR; %) at the highest tested concentrations and TPO inhibition (%) dose-response curves. These results demonstrate that the nonspecific impact of the chemicals on the fluorescence did not significantly affect the interpretation of TPO-inhibition potential in case of PFOS (nonactive) and TBBPA (active) and could only have a minor influence in the case of triclosan. Nevertheless, for triclosan the slight decrease in fluorescence could be related with the presence of observed precipitate in the RFR test without the cell lysate, which was not observed in the TPO-inhibition assay with the presence of lysate.

Figure S4. The dose-response effect of TCS in AUR assay conducted with HEK-TPOA7 lysate at elevated and normal pH level. Higher inhibition was detected at elevated pH levels. The total reaction volume is 100 µL in the AUR assay. For the higher pH, 100 µL total reaction volume contained 50 µL protein dilution, 45 µL GNE buffer AUR and 5 µL H_2_O_2_ with final pH 8.8. For the normal pH (7.2), 100 µL total reaction volume contained 50 µL protein dilution and 50 µL PBS with chemical and AUR.


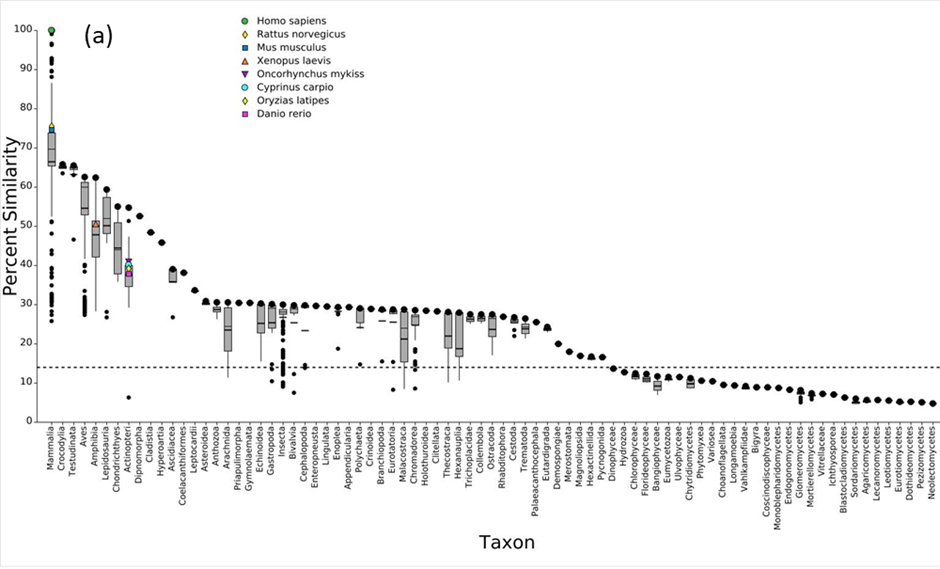

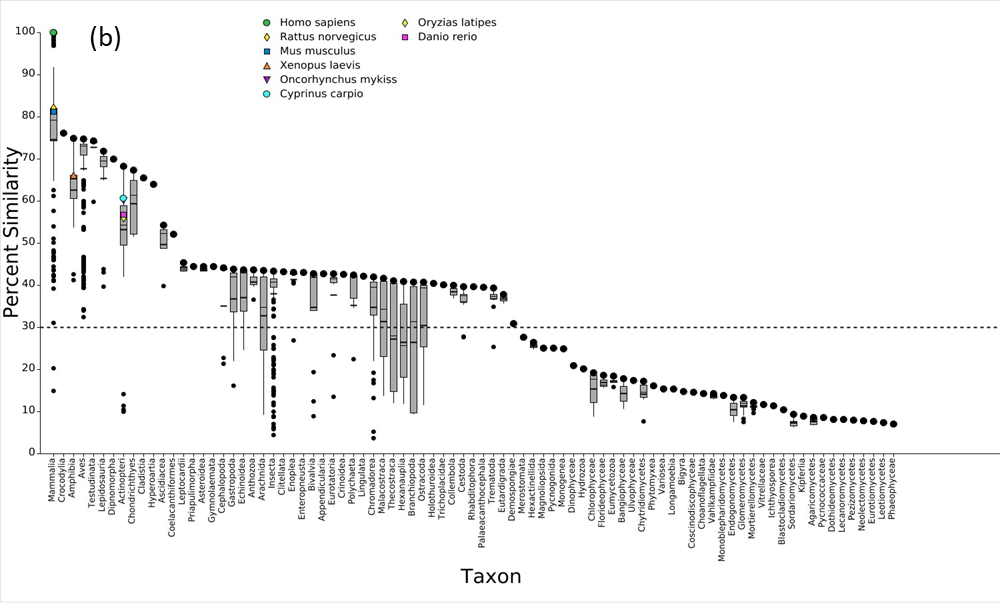


Figure S5. (a) SeqAPASS Level 1 visualization boxplot. (b) Level 2 visualization boxplot. The dotted line is the cut-off line, which was automatically determined by identifying the first ortholog candidate (human) at an equal or higher percent similarity than the first local minimum percent similarity. A detailed explanation can be found at https://www.epa.gov/comptox-tools/seqapass-user-guide.
